# The structural, functional, and therapeutic potential of metacaspases in fungi and protozoa

**DOI:** 10.1111/febs.70327

**Published:** 2025-11-12

**Authors:** Ane C. M. Duarte, Mariana N. R. Trujilho, Karolaine S. S. M. Valdivia, Aline F. Araujo, Laura M. C. de Lorena, Vinicius H. de Oliveira, Beatriz V. Pereira, Emilly R. Leme, Wagner A. S. Júdice, Maurício F. M. Machado

**Affiliations:** ^1^ Interdisciplinary Center for Biochemical Research University of Mogi das Cruzes Brazil

**Keywords:** calcium‐dependent proteases, fungal and protozoan pathogens, metacaspase, programmed cell death, proteostasis, self‐processing, therapeutical potential

## Abstract

Metacaspases are cysteine proteases found in fungi, protozoa, and plants, where they regulate critical cellular processes such as programmed cell death (PCD), cell cycle progression, and protein homeostasis. Although structurally related to caspases, metacaspases differ in mechanism of activation, substrate specificity, and biological roles. Unlike caspases, metacaspases are monomeric calcium‐dependent enzymes that cleave substrates after basic residues such as arginine or lysine. This review provides a comprehensive overview of the structural classification, biochemical regulation, and physiological functions of metacaspases in model eukaryotes. We discuss their roles in stress adaptation, cell death, and proteostasis in organisms such as *Saccharomyces cerevisiae*, *Candida albicans*, *Trypanosoma brucei*, and *Trypanosoma cruzi*. We highlight recent advances in understanding their activation via calcium binding and autocatalytic processing, and explore their functional diversity across species. In addition, we examine the therapeutic potential of metacaspases as drug targets due to their absence in mammals and essential roles in pathogenic microbes. Challenges in substrate identification, enzymatic characterization, and inhibitor design are also addressed, along with emerging tools that may accelerate metacaspase research. Altogether, this review underscores the growing importance of metacaspases in eukaryotic biology and their promising applications in antifungal and antiparasitic drug development.

## Graphical abstract

Metacaspases are Ca^2+^‐dependent cysteine proteases whose structural characteristics, particularly calcium binding and autocatalytic processing, govern their activation and biological functions. These enzymes differ among microorganisms and regulate their cell cycles, making them excellent targets for drug development.

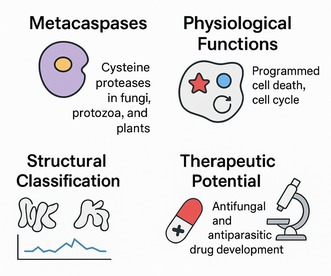

**Abbreviations**
CaMCA‐Ia: metacaspase of *C. albicans*
LmjMCA: metacaspase of *L. major*
PCD: programmed cell death
TbMCA: metacaspase of *T. brucei*
TcMCA: metacaspase of *T. cruzi*
TcoMCA5: metacaspase of *T. congolense*
YCA1: metacaspase of *S. cervisiae*

## Introduction

Metacaspases are proteolytic enzymes found in lower eukaryotic organisms such as fungi, protozoa, and plants, where they play fundamental roles in regulating processes including programmed cell death (PCD), stress response, and protein homeostasis [1, 2]. Despite structural similarities to mammalian caspases, metacaspases exhibit distinct biochemical properties, particularly in their substrate specificity, cleaving after basic residues such as arginine or lysine rather than aspartate, which is characteristic of caspases [2, 3].

The growing interest in metacaspases is fueled by advancements in molecular biology and structural biochemistry, which have enabled detailed studies on their catalytic specificity, activation mechanisms, and evolutionary conservation [4, 5]. Notably, metacaspases are absent in animals, including humans, which raises the prospect of exploiting them as potential therapeutic targets in parasitic infections [6, 7].

Proteases are classified by catalytic mechanism—based on the active site residue (e.g., serine, cysteine, threonine, aspartate, glutamate, or metal ion‐dependent)—and independently by cleavage position, as endopeptidases or exopeptidases [4, 8]. This distinction clarifies cases where enzymes share catalytic type but differ in cleavage sites, such as dipeptidyl peptidase‐1 and cathepsin L.

Cysteine proteases are widespread in eukaryotes and prokaryotes, where they regulate processes from growth to cell death. They share a catalytic dyad formed by cysteine and histidine residues [4].

These enzymes are also subdivided into clans according to their structure: in the CA clan (where most cysteine proteases are found), they are similar in the presence of the catalytic triad composed of Cys, His, and Asn residues. Examples of enzymes belonging to this clan are papain and proteases with structural similarity to it, called papain‐like. Cysteine proteases belonging to the CD clan, such as caspases in higher organisms and their analogs (metacaspases and paracaspases), have a catalytic dyad composed of Cys and His residues in their active site [9, 10].

The first cysteine peptidase biochemically isolated and characterized was papain, extracted from the latex of *Carica papaya* in 1937. This protease also pioneered the elucidation of its three‐dimensional structure through crystallography, in which it is possible to observe the presence of the catalytic triad composed of residues Cys^25^, His^159^, and Asn^175^ [9, 11, 12].

Substrate binding induces conformational changes that facilitate the hydrolysis reaction, and the precise mechanism depends on the protease class. Cysteine proteases, such as metacaspases, often utilize a nucleophilic attack by the cysteine residue activated by a neighboring histidine to cleave peptide bonds, forming a transient covalent intermediate [8].

The evolutionary grouping of proteases into families and clans is based on conserved structural motifs and catalytic mechanisms. Metacaspases, such as caspases, share a conserved tertiary fold and catalytic architecture but differ in activation and substrate processing, reflecting their divergent biological roles [3, 5].

## Structural classification and evolutionary aspects of metacaspases

Metacaspases are members of the cysteine protease class and share structural and catalytic features with other peptidases in the CD clan, notably the presence of a catalytic dyad formed by histidine and cysteine residues. According to the MEROPS database, metacaspases are categorized as the C14 family within the CD clan, which also includes caspases and paracaspases [4, 13].

The CD clan is characterized by a conserved tertiary structure formed by β‐sheets and α‐helices and by shared catalytic properties. Within this clan, the C14 family of proteases including caspases (in animals) and metacaspases (in fungi, protozoa, and plants) shows a common fold and catalytic mechanism but differs significantly in activation mode and substrate specificity [1, 5].

Structurally, metacaspases contain two primary subunits: a large catalytic domain known as P_20_, which includes the His/Cys dyad, and a smaller regulatory domain known as P_10_. Unlike caspases, which require dimerization and binding to adaptor molecules for activation, metacaspases are monomeric and can become active without forming complexes [14, 15]. Importantly, their activation is strictly calcium‐dependent, with Ca^2+^‐binding inducing conformational changes essential for substrate recognition and catalysis [2, 16].

**Fig. 1:**
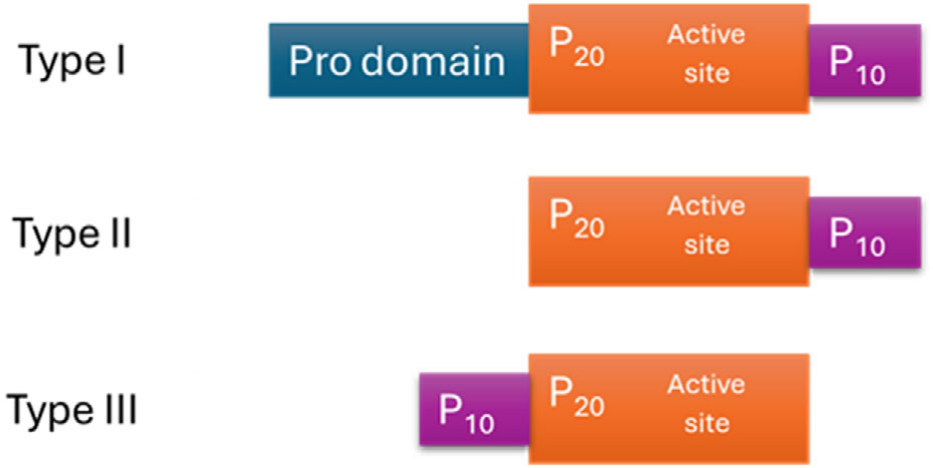
Comparison of different types of metacaspases. The schematic illustrates inactive metacaspase precursors (zymogens) contain an N‐terminal regulatory region and two conserved domains: the large catalytic domain (P_20_) and the small regulatory domain (P_10_). A type I metacaspase that contains a prodomain and P_20_ precedes P_10_, a type II metacaspase that is expressed without a pro domain, and a type III metacaspase that has the P_10_ unit preceding P_20_.

All metacaspases are α/β proteins that share a conserved α/β/α sandwich fold, within which calcium binding induces conformational rearrangements that expose the catalytic site and enable substrate access. They are divided into three main types [3] (Fig. 1).

The calcium‐binding sites are typically located in the P_20_ domain and are formed by conserved acidic residues, often aspartates, that coordinate calcium ions [15, 16, 17]. The presence of metacaspases across a wide range of nonmetazoan eukaryotes highlights their evolutionary significance. Their absence in mammals, combined with their conservation in fungi, protozoa, and plants, suggests that metacaspases represent an ancestral branch of cysteine proteases that diverged functionally from caspases [1, 3]. Phylogenetic analyses have shown that metacaspase genes are found in almost all eukaryotic lineages except for animals, reinforcing their distinct evolutionary trajectory [14].

Because of their structural and functional divergence, several experts have proposed a systematic revision of metacaspase nomenclature to reflect their unique biological roles. A consensus letter submitted to the MEROPS database curators in 2020 called for the adoption of a clear and consistent naming convention based on structural domains, catalytic activity, and evolutionary origin [3].

## Mechanisms of activation and catalytic regulation

Metacaspase activation is a tightly regulated process that differs significantly from that of mammalian caspases. While caspases are typically activated through dimerization and formation of large apoptosome complexes, metacaspases function as monomers and rely on distinct biochemical triggers most notably, calcium ions (Ca^2+^) to become enzymatically active [14, 15, 16, 18].

All metacaspases share a conserved catalytic dyad composed of a cysteine and a histidine residue, typically located within the P_20_ catalytic domain [1, 15, 17]. The histidine functions as a general base to activate the cysteine thiol group, enabling a nucleophilic attack on the peptide bond of the substrate. This mechanism is like that seen in other cysteine proteases but is regulated differently in metacaspases.

Metacaspases require calcium for activation, which induces conformational rearrangements that stabilize the catalytic site. Structural studies in YCA1 and TbMCA2 identified conserved acidic residues (e.g., Asp^236^, Asp^252^, Asp^283^) that coordinate Ca^2+^ and promote activity [15, 16, 17, 19].

The calcium‐binding sites are generally located near or within the P_20_ domain, and coordination of Ca^2+^ appears to induce rearrangements that enable proper orientation of the catalytic dyad, possibly by stabilizing flexible loops that surround the active site [15, 17]. Several metacaspases undergo autoprocessing a form of intramolecular cleavage that results in the removal of N‐terminal or C‐terminal regions, thereby exposing or stabilizing the catalytic site. For instance, YCA1 from *S. cerevisiae* is known to cleave itself at multiple positions in the presence of Ca^2+^, including after residues Arg^72^, Lys^86^, Lys^331^, and Lys^334^, generating active fragments of ~ 45, 31, and 12 kDa [15]. This autoprocessing enhances the catalytic efficiency of the enzyme, enabling it to process both oligopeptides and larger substrates.

Interestingly, the autoprocessing of YCA1 is not essential for catalytic activity *per se* but appears to contribute to the enzyme's ability to recognize and cleave full‐length protein substrates more efficiently [15]. This observation supports a two‐step activation model in which an initial calcium‐dependent conformational change primes the enzyme, followed by autoprocessing that broadens substrate specificity [16, 20].

Similar behavior has been reported in other metacaspases. For example, TcoMCA5 from *Trypanosoma congolense* undergoes spontaneous autoprocessing when expressed recombinantly in *E. coli*, indicating that structural rearrangement and intermolecular cleavage may also contribute to activation *in vivo* [21].

Unlike caspases, metacaspases do not require dimerization or assembly into high‐order complexes for activation [14]. However, studies have shown that once a metacaspase is activated (e.g., by Ca^2+^ and autoprocessing), it can participate in intermolecular processing of other inactive metacaspase molecules in its vicinity. This process potentially amplifies the cellular metacaspase signal and increases overall proteolytic activity [15, 19].

**Fig. 2:**
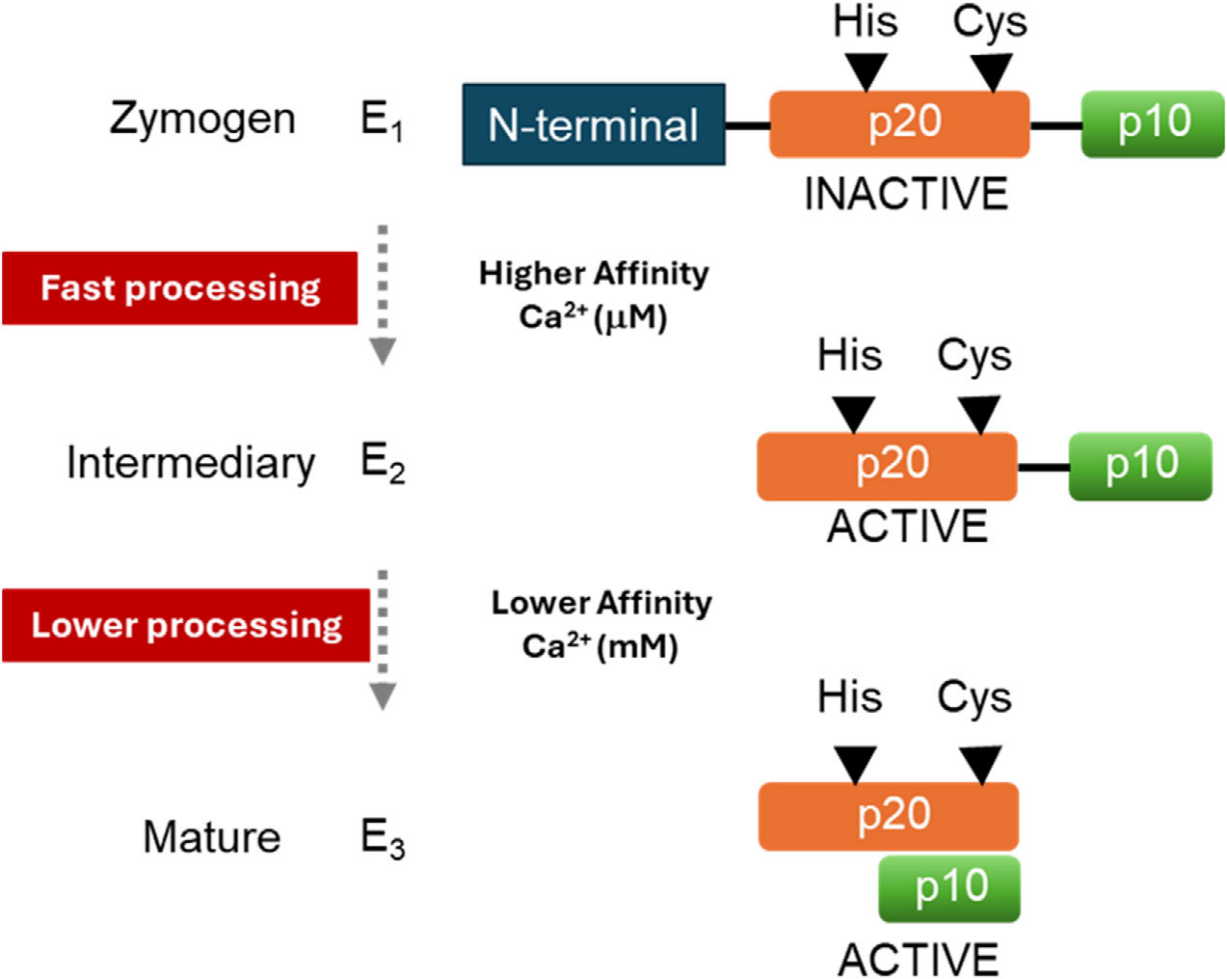
Illustrative representation proposed for metacaspase during its autoprocessing. Metacaspases are Ca^2+^‐dependent cysteine proteases found in fungi and protozoa but absent in mammals. The figure illustrates the general domain organization of metacaspases, including the large catalytic subunit (P_20_) containing the histidine (His) and cysteine (Cys) catalytic dyad and the smaller regulatory subunit (P_10_). Calcium ion binding and autocatalytic processing at basic residues such as arginine (Arg) and lysine (Lys) trigger conformational rearrangements that activate the enzyme. Once active, metacaspases cleave substrates after Arg or Lys residues, regulating essential cellular processes such as programmed cell death (PCD), cell cycle progression, and protein homeostasis (proteostasis). The schematic also emphasizes their absence in animals and their potential as selective therapeutic targets in antifungal and antiparasitic drug development.

Experimental evidence from *Candida glabrata* metacaspases suggests that enzymes exist in two affinity states: a ‘low‐affinity’ unprocessed form and a ‘high‐affinity’ cleaved form. The high‐affinity version is more catalytically active and can cleave other metacaspase molecules, thereby converting them to the active state (Fig. 2) [22]. The same data were observed for metacaspases of *Saccharomyces cerevisiae* (YCA1) and *Candida albicans* (CaMCA‐Ia), showing that it is a common comportment for metacaspases of yeast [19, 23].

Some metacaspases, particularly those of Type I, possess N‐terminal prodomains that regulate access to the active site. In *Trypanosoma brucei*, structural studies of TbMCA2 showed that the N‐terminal region may act as an autoinhibitory segment by covering the substrate‐binding cleft. Removal of this segment through autoprocessing or conformational change enables catalytic function [17, 18, 20].

Similarly, in *T. cruzi*, the metacaspase TcMCA5 contains a C‐terminal domain that acts as a negative regulator of catalytic activity. Deletion of this domain enhances proteolytic activity and increases the enzyme's ability to induce programmed cell death [24].

Taken together, these findings highlight the complex, multilayered regulation of metacaspase activation. Calcium dependence, structural rearrangements, autoprocessing, and domain occlusion together define a unique model of protease regulation that is both structurally and mechanistically distinct from that of caspases.

## Biological functions of metacaspases

### Programmed cell death (PCD)

Although metacaspases do not possess caspase‐like specificity for aspartate residues, they have been extensively associated with apoptosis‐like processes in lower eukaryotes. Several lines of evidence suggest that metacaspases are involved in cell death pathways that share morphological and biochemical features with apoptosis, such as chromatin condensation, DNA fragmentation, and increased production of reactive oxygen species [14, 25, 26, 27].

In the yeast *Saccharomyces cerevisiae*, the metacaspase YCA1 was the first to be directly linked to regulated cell death. YCA1 deletion mutants were shown to be more resistant to oxidative stress induced by hydrogen peroxide (H_2_O_2_), indicating that the presence of YCA1 promotes cell death under stress conditions [26]. Overexpression of YCA1, in contrast, exacerbates the death phenotype, further reinforcing its prodeath role.

In *Candida albicans*, a pathogenic yeast, the metacaspase CaMCA is also activated in response to oxidative stress. Treatments with H_2_O_2_, acetic acid, or amphotericin B lead to hallmarks of apoptosis, including mitochondrial dysfunction and chromatin fragmentation, which are accompanied by increased CaMCA activity [27]. Inhibition of CaMCA using specific inhibitors or genetic approaches has been shown to reduce the extent of programmed cell death, confirming its functional involvement.

Whether fungi undergo apoptosis‐like PCD remains debated. Hardwick [28] argues that applying the term ‘apoptosis’ is misleading, since fungi lack caspase‐3 and instead encode structurally distinct metacaspases. Until molecular effectors are identified, fungal PCD should be described cautiously, with alternative terms such as ‘mycoptosis’ proposed. Emerging data also suggest parallels with necroptosis, ferroptosis, and autophagy‐dependent cell death [28].

Similarly, in protozoan parasites such as *Leishmania major*, the metacaspase LmjMCA is cleaved during stress responses, releasing a catalytically active fragment that translocates to the cytoplasm and triggers cell death [29, 30, 31]. The cleavage of LmjMCA appears to be regulated by environmental cues and is linked to parasite differentiation and pathogenesis.

In *Plasmodium falciparum*, the causative agent of malaria, metacaspases have also been implicated in cell death. PfMCA2, one of the metacaspases identified in this parasite, was shown to cleave Tudor Staphylococcal Nuclease (TSN), a protein involved in RNA metabolism. TSN cleavage disrupts essential cellular processes, thereby promoting parasite [32].

Notably, the exact molecular pathways through which metacaspases mediate cell death remain unclear. Unlike mammalian caspases, which are components of well‐defined apoptotic cascades, metacaspases appear to function as part of organism‐specific PCD mechanisms, often lacking clear homologs to initiator or effector proteins found in animals [3, 33, 34]. Furthermore, while calcium is essential for metacaspase activation, its role in signaling cell death pathways in fungi and protozoa is still being elucidated.

Despite these gaps, it is now widely accepted that metacaspases play a pivotal role in orchestrating cell death under certain stress conditions in lower eukaryotes. This function may be adaptive, allowing organisms to eliminate damaged cells, regulate population density, or respond to environmental pressures such as nutrient deprivation or host immune response [35].

### Cell cycle regulation

In addition to their role in cell death, metacaspases have emerged as important regulators of the cell cycle in various unicellular eukaryotes. This regulatory function appears to be closely linked to their proteolytic activity, which targets specific substrates involved in the progression and control of cell division [36].

In *Saccharomyces cerevisiae*, the metacaspase YCA1 has been shown to contribute to mitotic fidelity and genome stability. Although deletion of YCA1 does not impair cell viability under normal conditions, it results in increased accumulation of protein aggregates and chromosomal instability during replicative aging [36]. This phenotype suggests a role for YCA1 in quality control mechanisms that preserve cellular homeostasis throughout the cell cycle.

Experimental evidence also indicates that YCA1 interacts with cell cycle‐regulated substrates, although its precise targets remain under investigation. One proposed mechanism involves the degradation of oxidized or misfolded proteins that accumulate during cell cycle progression, particularly in aging or stressed cells. This degradative activity prevents the interference of damaged proteins with mitotic machinery and ensures proper division [35, 37].

In *Candida albicans*, CaMCA activity is modulated throughout the cell cycle and is upregulated in response to stress conditions that impair cell division. Exposure to high concentrations of calcium or oxidative agents leads to an increase in CaMCA activity, coinciding with growth arrest and morphological changes such as filamentation [27, 38]. These findings suggest that metacaspase activation in fungi is integrated into stress surveillance pathways that coordinate cell cycle checkpoints with environmental cues.

In protozoan parasites, particularly *Trypanosoma brucei*, metacaspase TbMCA2 exhibits tightly regulated expression during different cell cycle stages. Studies have demonstrated that silencing of TbMCA2 leads to an increase in cells arrested at the G2/M boundary, indicating a role in facilitating the transition into mitosis [39]. This may involve cleavage of yet‐unidentified substrates that participate in cytoskeletal reorganization or nuclear division.

A related mechanism has been proposed for *Leishmania major*, where metacaspase activity correlates with parasite differentiation a process accompanied by significant remodeling of cell architecture and division mode. The ability of metacaspases to cleave cytoskeletal or regulatory proteins during these stages may allow the fine‐tuning of the cell cycle in response to environmental or developmental signals [29, 30].

Altogether, these observations indicate that metacaspases are more than simple executioners of cell death; they also contribute to the temporal and spatial coordination of cell division in lower eukaryotes. This dual function is particularly relevant in pathogenic organisms, where the ability to switch between proliferation and dormancy or differentiation states is essential for survival and virulence [3, 5].

### Proteostasis and protein quality control

Beyond their established roles in cell death and cell cycle regulation, metacaspases have gained recognition for their involvement in maintaining protein homeostasis, or proteostasis, particularly under stress conditions or during aging. This function is essential for the removal of damaged or aggregated proteins, preventing proteotoxic stress and preserving cellular viability [35, 36].

In *Saccharomyces cerevisiae*, deletion of the YCA1 gene leads to the accumulation of protein aggregates during replicative aging, suggesting that this metacaspase plays a central role in proteostasis maintenance [36]. The enzymatic activity of YCA1 contributes to the degradation of oxidatively damaged or misfolded proteins that arise naturally during cellular metabolism or in response to oxidative stress.

Further studies have shown that YCA1 is functionally integrated into cellular systems for protein quality control. For example, its activity is coordinated with molecular chaperones and components of the ubiquitin‐proteasome system, reinforcing the view that metacaspases participate in selective degradation pathways aimed at removing defective proteins [35, 37]. This function becomes particularly critical during replicative and chronological aging, when the accumulation of damaged proteins poses a major threat to cellular integrity.

Interestingly, YCA1 has been implicated in the asymmetric inheritance of protein aggregates during yeast cell division. In this model, YCA1 selectively degrades aggregation‐prone proteins in the mother cell, ensuring that daughter cells are born free of toxic aggregates. This contributes to rejuvenation in daughter cells and helps extend replicative lifespan [35, 36].

The role of metacaspases in proteostasis is not restricted to yeasts. In *Candida albicans*, the metacaspase CaMCA also contributes to the clearance of stress‐induced protein aggregates, particularly following exposure to oxidative or thermal stress [38, 40, 41]. CaMCA‐deficient mutants exhibit higher sensitivity to such stressors and show a reduced ability to recover from protein‐damaging insults.

Similarly, in protozoan parasites such as *Trypanosoma brucei*, metacaspases are upregulated during environmental stress and may participate in the degradation of damaged proteins accumulated during host infection or drug exposure. Although the specific substrates involved in these processes remain to be identified, evidence suggests that metacaspases act in concert with other proteolytic systems to modulate the parasite's proteome during critical transitions [39, 42].

These findings point to a broader functional scope for metacaspases as adaptive enzymes capable of monitoring and modulating the intracellular proteome. By targeting oxidized or misfolded proteins for degradation, metacaspases help maintain proteome integrity, prevent aggregation, and restore homeostasis—especially in long‐lived or highly adaptive unicellular organisms [3, 5].

## Case studies in model eukaryotes

The YCA1 gene of *Saccharomyces cerevisiae* encodes the first metacaspase to be functionally characterized and remains the most extensively studied example among fungi. YCA1 is a Type I metacaspase and serves as a central model for understanding the structural, biochemical, and physiological properties of this protease family [15, 25].

YCA1 is composed of 432 amino acids and features the canonical His‐Cys catalytic dyad located in the P_20_ domain. The N‐terminal region, rich in proline, glutamine, and asparagine residues, acts as a regulatory domain and is not essential for catalytic activity [15].

Recombinant expression of full‐length and N‐terminally truncated forms of YCA1 in bacterial systems has allowed detailed enzymatic characterization. These studies demonstrated that both versions are catalytically active in the presence of calcium and preferentially cleave after arginine residues [2, 15]. Site‐directed mutagenesis confirmed the essential role of His^171^ and Cys^220^ in catalysis, as substitution of these residues abolishes enzymatic activity [15].

YCA1 undergoes autocatalytic processing in response to calcium, with cleavage sites identified at Lys^86^ (N terminus) and Lys^331^ or Lys^334^ (C terminus). The partially processed form retains catalytic activity, although with different efficiency depending on the level of cleavage [15]. This calcium‐dependent activation mechanism distinguishes YCA1 from caspases, which require dimerization and adaptor‐mediated activation.

Functionally, YCA1 plays roles in multiple cellular processes, including PCD, cell cycle regulation, and proteostasis. Under oxidative stress conditions, such as hydrogen peroxide exposure, YCA1 becomes activated and promotes apoptotic‐like features in yeast cells [25]. Conversely, YCA1‐deficient strains exhibit resistance to stress‐induced cell death and accumulate damaged proteins during aging, reinforcing its role in protein quality control [35, 36, 37].

Localization studies have shown that YCA1 is predominantly cytosolic but may transiently associate with mitochondria under stress, potentially linking its activity to mitochondrial dysfunction during apoptosis [25]. Additionally, YCA1 contributes to asymmetric inheritance of protein aggregates during cell division, preserving daughter cell vitality [35]. Overall, YCA1 represents a prototypical metacaspase whose structural features, catalytic regulation, and multifunctional roles provide important insights into the broader metacaspase family in eukaryotic microbes.

In the opportunistic pathogenic yeast *Candida albicans*, the metacaspase CaMCA has been identified as a Type I metacaspase that plays key roles in programmed cell death, morphogenesis, and adaptation to environmental stress. Its characterization has expanded the understanding of fungal metacaspases beyond the model organism *Saccharomyces cerevisiae* and highlighted its relevance in virulence and antifungal response [38, 43, 44, 45].

CaMCA shares the typical structural organization of Type I metacaspases, with an N‐terminal proline/glutamine/asparagine‐rich region, followed by the P_20_ catalytic domain and a short C‐terminal P_10_ domain. Like other metacaspases, CaMCA contains the conserved His‐Cys catalytic dyad and exhibits calcium‐dependent activation [16, 27].

Functional studies have demonstrated that CaMCA is activated in response to oxidative stressors such as hydrogen peroxide, acetic acid, and antifungal agents including amphotericin B [27]. Activation of CaMCA under these conditions leads to apoptotic‐like features including chromatin condensation, DNA fragmentation, and externalization of phosphatidylserine. In CaMCA‐deficient strains, these apoptotic phenotypes are significantly attenuated, indicating that CaMCA is a key mediator of cell death in *C. albicans*.

Like YCA1 in *S. cerevisiae*, CaMCA is also involved in regulating protein homeostasis and cell cycle progression. Its activation correlates with increased clearance of stress‐induced protein aggregates, and its absence leads to higher sensitivity to heat and oxidative stress [36, 46]. These findings support a conserved role for fungal metacaspases in maintaining proteome stability during environmental challenges.

Importantly, CaMCA appears to be involved in the morphogenetic transition of *C. albicans* from yeast to hyphal forms a key process associated with pathogenicity. Disruption of CaMCA impairs filamentation under inducing conditions, suggesting a link between metacaspase activity and morphological differentiation [47]. This connection underscores the potential of CaMCA as a target for antifungal therapies aimed at reducing virulence.

Taken together, CaMCA represents a multifunctional metacaspase with roles in apoptosis‐like death, stress adaptation, and morphogenesis. Its characterization reinforces the concept that fungal metacaspases integrate environmental signals with intracellular processes to coordinate adaptive responses and pathogenic potential.

In the protozoan parasite *Trypanosoma brucei*, the causative agent of African trypanosomiasis, metacaspases play vital roles in stress response, cell cycle regulation, and parasite viability. The genome of *T. brucei* encodes five metacaspase genes (TbMCA1–5), but among them, TbMCA2 and TbMCA3 have been most extensively characterized due to their robust enzymatic activity and clear biochemical properties [16, 39, 48].

TbMCA2 is a Type II metacaspase and differs structurally from fungal Type I metacaspases by lacking the N‐terminal prodomain and instead featuring a longer interdomain linker between the P_20_ and P_10_ regions. TbMCA2 is synthesized as a zymogen and undergoes Ca^2+^‐dependent autocatalytic cleavage to become active. This processing occurs preferentially after basic residues such as Lys^55^ and Lys^268^ and results in an enzymatically active form that retains substrate specificity for arginine at the P1 position [16, 17, 20].

Biochemical analyses of recombinant TbMCA2 have shown that its activity depends on millimolar concentrations of calcium, which induces a conformational rearrangement that exposes the catalytic site [20]. Site‐directed mutagenesis targeting calcium‐binding residues within the P_20_ domain significantly impairs enzymatic activity, confirming that calcium plays a structural and regulatory role, not merely an allosteric one [16].

The functional relevance of TbMCA2 extends to parasite viability and cell division. RNA interference‐mediated silencing of TbMCA2 leads to cell cycle arrest at the G2/M phase, indicating its involvement in mitotic progression. Additionally, TbMCA2 may be implicated in the degradation of misfolded or aggregated proteins during host adaptation, although its specific substrates remain unidentified [39, 49]. Together, TbMCA2 and its homologs serve as valuable models for understanding the nonapoptotic roles of metacaspases in protozoa and highlight their potential as therapeutic targets in the treatment of trypanosomiasis.

*Trypanosoma cruzi*, the etiologic agent of Chagas disease, possesses two metacaspase genes in its genome, designated TcMCA3 and TcMCA5. These metacaspases are less characterized than their counterparts in *T. brucei*, but emerging evidence suggests that they participate in essential biological processes such as parasite differentiation, adaptation to environmental stress, and possibly programmed cell death [24, 50, 51].

TcMCA3 and TcMCA5 exhibit structural features typical of Type I metacaspases, including an N‐terminal extension and the conserved catalytic His‐Cys dyad. Despite this, recombinant expression studies have shown that their enzymatic activity is relatively low under standard assay conditions, suggesting a requirement for additional regulatory mechanisms or post‐translational modifications for full activation [51].

Unlike TbMCA2 in *T. brucei*, TcMCAs are not readily activated by calcium alone, which has made their biochemical characterization more challenging. It is possible that their activation depends on specific intra‐ or intermolecular processing events that have not yet been fully elucidated [50].

Inhibition of metacaspase activity in *T. cruzi* results in altered morphology, reduced infectivity, and impaired differentiation, further supporting a physiological role for these enzymes [50].

## Therapeutic potential of metacaspases

The absence of metacaspases in mammals, along with their essential roles in the physiology of fungi and protozoan parasites, makes them attractive targets for therapeutic intervention. Their involvement in programmed cell death, cell cycle regulation, and stress adaptation in pathogenic organisms suggests that modulation of metacaspase activity could interfere with parasite survival and virulence [6, 52].

In fungal pathogens such as *Candida albicans*, metacaspases have been linked to the regulation of apoptosis‐like death following treatment with antifungal agents, including amphotericin B. Pharmacological inhibition or genetic deletion of the CaMCA gene reduces the apoptotic response and improves survival under antifungal stress, suggesting that metacaspase activity sensitizes cells to drug‐induced cell death [27]. This finding opens avenues for the development of adjuvant therapies that potentiate current antifungal agents by manipulating metacaspase pathways.

In trypanosomatid protozoa, particularly *Trypanosoma brucei* and *T. cruzi*, metacaspases are implicated in processes essential to parasite viability and adaptation, such as differentiation, protein homeostasis, and cell cycle progression [7, 20, 53]. Importantly, silencing or mutagenesis of specific metacaspase genes in these parasites disrupts normal cellular functions and may reduce infectivity. This provides a rationale for exploring small molecule inhibitors that selectively target parasite metacaspases while sparing host proteases.

The structural differences between metacaspases and human caspases further enhance their appeal as drug targets. Metacaspases cleave substrates after basic residues (arginine or lysine), whereas caspases cleave after aspartate residues. Additionally, metacaspases rely on calcium for activation, a feature absent in caspases. These distinct characteristics offer opportunities for designing highly selective inhibitors based on substrate‐mimicking scaffolds or metal‐chelating strategies [3].

Efforts to identify metacaspase inhibitors have included the use of broad‐spectrum cysteine protease inhibitors, as well as the rational design of substrate analogs that mimic arginine‐containing cleavage sites. Although no specific metacaspase‐targeted drugs have yet reached clinical use, the feasibility of designing such compounds has been supported by structural data from crystallographic and mutational studies [7, 16, 50, 54].

The potential for targeting metacaspases is further strengthened by their expression profiles during infection. In *T. brucei*, metacaspase expression is developmentally regulated, with elevated levels during differentiation from bloodstream to procyclic forms. This suggests that therapeutic modulation of metacaspase activity could interfere with the parasite life cycle at critical stages [39]. Similarly, in *C. albicans*, metacaspase activity is linked to morphogenetic transitions and virulence traits, which could be exploited to attenuate pathogenicity [27, 38].

Nonetheless, several challenges remain. The redundancy of metacaspase isoforms in some organisms, the partial overlap with other proteolytic systems, and the potential for off‐target effects must be considered in the development of selective inhibitors. In addition, the lack of high‐throughput screening platforms specifically optimized for metacaspase activity has limited progress in drug discovery.

Despite these obstacles, the unique biochemical features and pathogen‐specific functions of metacaspases justify continued exploration of their therapeutic potential. Advances in structure‐guided drug design, coupled with functional genomics in pathogenic fungi and protozoa, are expected to accelerate the identification of viable metacaspase‐targeting compounds.

## Conclusions

Metacaspases constitute a structurally and functionally diverse family of cysteine proteases that play crucial roles in nonmetazoan eukaryotes, including fungi and protozoan parasites. Although initially identified due to their structural resemblance to caspases, metacaspases have since been recognized as distinct enzymes with unique regulatory mechanisms, calcium‐dependent activation, and substrate specificities that target basic residues rather than aspartate.

Studies in model organisms such as *Saccharomyces cerevisiae*, *Candida albicans*, *Trypanosoma brucei*, and *T. cruzi* have revealed multifaceted functions for metacaspases, ranging from programmed cell death and stress adaptation to cell cycle regulation and protein quality control. These activities are tightly integrated into organism‐specific pathways that govern survival, differentiation, and pathogenicity.

Despite significant progress, key aspects of metacaspase biology remain poorly understood, including their full repertoire of physiological substrates, the precise mechanisms underlying activation and regulation, and their broader roles within cellular networks. Overcoming these challenges will be essential for translating basic insights into practical applications.

Importantly, the absence of metacaspases in animals combined with their essential roles in the viability and virulence of microbial pathogens positions them as promising targets for therapeutic development. Future studies that integrate biochemical, genetic, structural, and pharmacological approaches are likely to yield not only deeper mechanistic understanding but also novel strategies to combat fungal and parasitic infections.

In conclusion, metacaspases represent an evolutionarily conserved but mechanistically distinct branch of the cysteine protease family, whose full biological relevance and therapeutic potential are just beginning to be uncovered.

## Conflict of interest

The authors declare no conflict of interest.

## Author contributions

KSSMV, AFA, LMCL, VHO, BVP, and ERL carried out the bibliographic research and the first data tracking; ACMD and MNRT compiled the data and wrote the first version of the review and MFMM and WASJ wrote and revised the final version.
